# Consistent Stool Metagenomic Biomarkers Associated with the Response To Melanoma Immunotherapy

**DOI:** 10.1128/msystems.01023-22

**Published:** 2023-02-21

**Authors:** Evgenii I. Olekhnovich, Artem B. Ivanov, Anna A. Babkina, Arseniy A. Sokolov, Vladimir I. Ulyantsev, Dmitry E. Fedorov, Elena N. Ilina

**Affiliations:** a Lopukhin Federal Research and Clinical Center of Physical-Chemical Medicine of Federal Medical Biological Agency, Moscow, Russian Federation; b ITMO University, Saint Petersburg, Russian Federation; c Moscow Institute of Physics and Technology, Moscow, Russian Federation; Vall d’Hebron Institut de Recerca

**Keywords:** gut microbiota, cancer immunotherapy, melanoma, metagenomics, fecal transplantation, compositional data analysis

## Abstract

The human gut microbiome plays an important role in both health and disease. Recent studies have demonstrated a strong influence of the gut microbiome composition on the efficacy of cancer immunotherapy. However, available studies have not yet succeeded in finding reliable and consistent metagenomic markers that are associated with the response to immunotherapy. Therefore, the reanalysis of the published data may improve our understanding of the association between the composition of the gut microbiome and the treatment response. In this study, we focused on melanoma-related metagenomic data, which are more abundant than are data from other tumor types. We analyzed the metagenomes of 680 stool samples from 7 studies that were published earlier. The taxonomic and functional biomarkers were selected after comparing the metagenomes of patients showing different treatment responses. The list of selected biomarkers was also validated on additional metagenomic data sets that were dedicated to the influence of fecal microbiota transplantation on the response to melanoma immunotherapy. According to our analysis, the resulting cross-study taxonomic biomarkers included three bacterial species: Faecalibacterium prausnitzii, Bifidobacterium adolescentis, and Eubacterium rectale. 101 groups of genes were identified to be functional biomarkers, including those potentially involved in the production of immune-stimulating molecules and metabolites. Moreover, we ranked the microbial species by the number of genes encoding functionally relevant biomarkers that they contained. Thus, we put together a list of potentially the most beneficial bacteria for immunotherapy success. *F. prausnitzii*, E. rectale, and three species of bifidobacteria stood out as the most beneficial species, even though some useful functions were also present in other bacterial species.

**IMPORTANCE** In this study, we put together a list of potentially the most beneficial bacteria that were associated with a responsiveness to melanoma immunotherapy. Another important result of this study is the list of functional biomarkers of responsiveness to immunotherapy, which are dispersed among different bacterial species. This result possibly explains the existing irregularities between studies regarding the bacterial species that are beneficial to melanoma immunotherapy. Overall, these findings can be utilized to issue recommendations for gut microbiome correction in cancer immunotherapy, and the resulting list of biomarkers might serve as a good stepping stone for the development of a diagnostic test that is aimed at predicting patients’ responses to melanoma immunotherapy.

## INTRODUCTION

Over the past decades, the main cancer treatment options were surgery, ionizing radiation, and chemotherapy. However, recently, novel approaches of treating melanoma and other cancerous types emerged. Since these approaches are based on the activation of patients’ own immunities, they collectively became known as immunotherapy. The therapeutic effects are achieved through the administration of immune checkpoint inhibitors (ICIs), which induce a T-lymphocyte-mediated immune response against tumors (1). However, responses to these treatments are notoriously heterogeneous and nondurable. A significant number of patients fail to benefit from ICIs (2), whereas others exhibit severe autoimmune side effects (3).

The gut microbiome takes part in metabolic reactions that are utterly important for human health. Human studies have reported differences in the gut microbiota compositions of cancer patients who exhibited different responses to immunotherapy (RI) (4–10). Moreover, the transplantation of feces from a responder patient may modulate the outcome of a nonresponder patient (11, 12). These experiments confirmed the crucial role of the gut microbiome in determining the efficacy of immunotherapy. The development of this line of research may lead not only to the creation of new diagnostic tools but also, possibly, to new medications and treatment strategies that are aimed toward increasing the effectiveness of immunotherapy. However, despite the large number of studies that have been published, researchers still have not reached a consensus on the gut microbial determinants of responsiveness to melanoma immunotherapy (RMI). Moreover, the published meta-analyses (13, 14) do not provide a clear answer to this question. While there is no doubt that the frequently cited objective factors, such as the complexity of metagenomic data, technical and/or biological variations, as well as specific individual factors, partially explain the lack of consistent results, some data analysis strategies may be better than others for revealing previously unidentified trends and dependencies.

In this study, we identified consistent biomarkers that are associated with positive ICIs therapy outcomes by applying compositional data analysis methods (15) to stool metagenomic data from earlier published studies. We also focused on melanoma-related data, as these are more abundant than are data related to other tumor types, and we improved the accuracy of our analysis by employing the approaches that we developed earlier, including MetaCherchant (16) and Recipient intestinE Colonization AnalysiS Tool (RECAST) (17). As a result, consistent stool metagenomic biomarkers that are associated with RMI were identified by comparing the stool samples of patients who displayed different treatment responses. We also validated our findings on available data sets that were dedicated to the impact of fecal microbiota transplantation on RMI as well as on other metagenomic data sets. Additionally, we established connections between taxonomic and functional biomarkers that allowed us to single out the potentially most beneficial bacteria that possibly contribute to RMI and to verify the results of previous analyses.

## RESULTS

### Discovery of consistent taxonomic biomarkers associated with RI.

The aim of our study was to find consistent taxonomic biomarkers that are associated with RI. Thus, the metagenomic sequencing data of 358 stool samples from 5 studies that are available in the NCBI database were included in the analysis: the group comparison of melanoma patient stool samples with different RI outcomes (Frankel 2017, Gopalakrishnan 2018, and Matson 2018; here, called the group 1 data sets; 47 responders [R] versus 55 nonresponders [NR]; 102 metagenomes) as well as the data analysis results of the fecal microbiota transplantation influence on RI (Baruch 2021 and Davar 202; here, called the group 2 data sets; 9 donors; 6 responders; 19 nonresponders; 256 metagenomes). The sequencing statistics and other metadata that characterize the metagenomes are presented in Table S1.

10.1128/msystems.01023-22.5 TABLE S1 Summary data and sequencing statistics of the metagenomes included in the analysis. Download Table S1, XLSX file, 0.02 MB. Copyright © 2023 Olekhnovich et al. 2023 Olekhnovich et al. https://creativecommons.org/licenses/by/4.0/ This content is distributed under the terms of the Creative Commons Attribution 4.0 International license.

**FIG 1:**
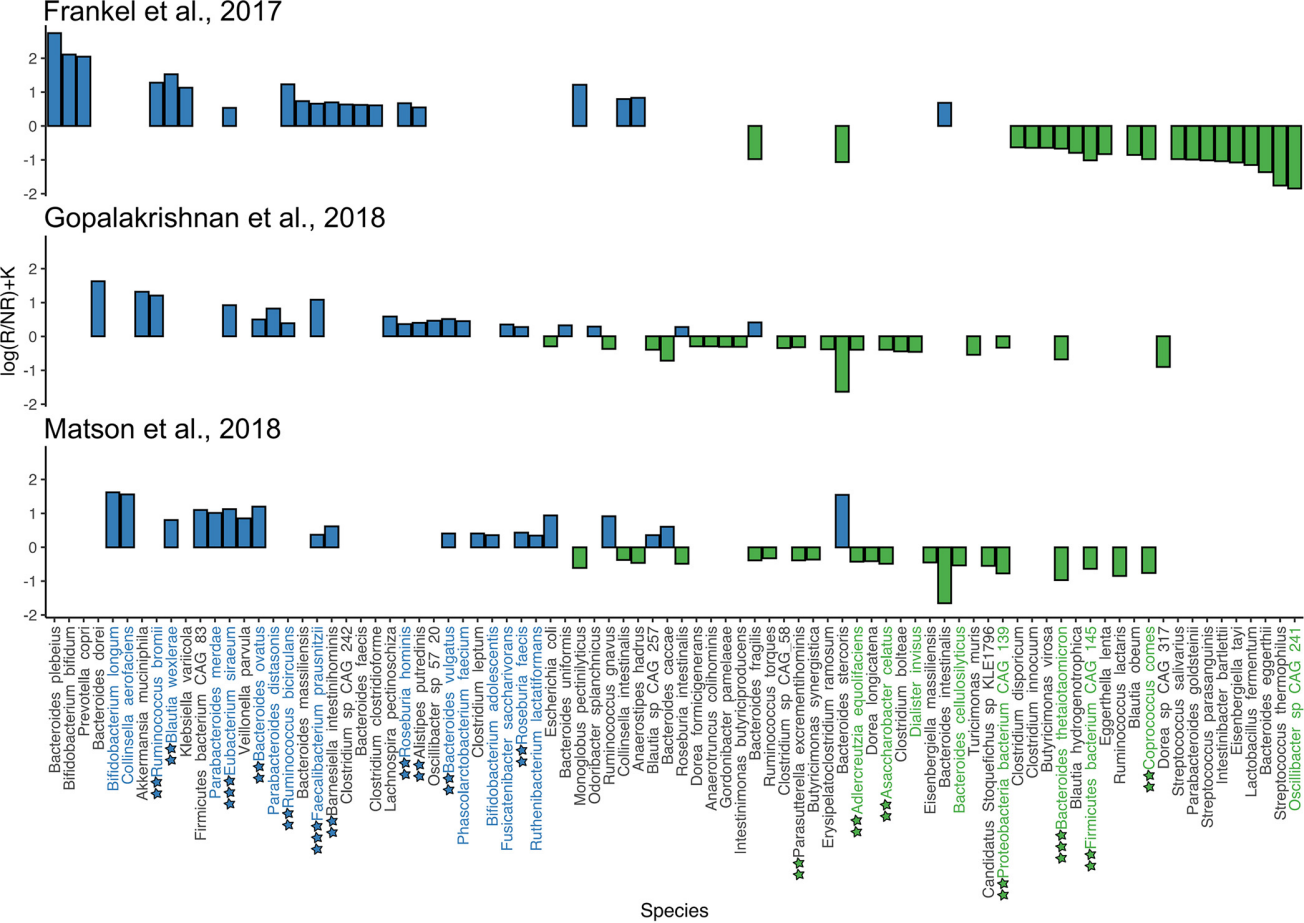
Species rankings and differentials generated by Songbird. Species are plotted on the *x* axis, and differentials describing the log-fold change in taxa associated with RI are plotted on the *y* axis. Positive coefficient levels correspond to RMI, and negative levels correspond to an unresponsiveness to melanoma immunotherapy (UMI). Two colored stars designate species associated with RMI and UMI in two data sets, and three stars designate species associated with RMI and UMI in three data sets. Species that contain consistent biomarkers are highlighted in blue and green.

The search for consistent taxonomic biomarkers that are associated with RMI was carried out in two steps. At the first stage, the taxa of the gut metagenomes that set R apart from NR were identified by applying the differential rankings approach via Songbird individually for the data sets in Group 1. The top 20 taxa displaying both positive and negative differential values were selected for further analysis. The log-ratio assessment of the selected taxa across metagenomes shows a clear, statistically significant difference between the R and NR groups (Fig. S1). The concatenated Songbird-derived rankings and differentials are illustrated in Fig. 1. According to our findings, nine bacterial species (Faecalibacterium prausnitzii, Eubacterium siraeum, Ruminococcus bromii, Blautia wexlerae, Bacteroides ovatus, Ruminococcus bicirculans, Barnesiella intestinihominis, Roseburia hominis, Alistipes putredinis, Bacteroides vulgatus, and Roseburia faecis) were predictors of RMI in at least two data sets of group 1. In contrast, seven bacterial species were identified to be predictors of UMI: Bacteroides thetaiotaomicron, Parasutterella excrementihominis, Adlercreutzia equolifaciens, Asaccharobacter celatus, Proteobacteria bacterium CAG 139, Firmicutes bacterium CAG 145, and Coprococcus comes.

10.1128/msystems.01023-22.1 FIG S1 Feature rankings and differentials generated by Songbird (A, C, E) as well as boxplots of the log ratios of the selected features (B, D, F). The top 20 taxonomic features with both positive and negative differential values were selected for the analysis. The selected species that were subjected to the log ratios assessment are highlighted in blue and green colors, corresponding to the R and NR groups, respectively. The log-ratio statistical assessment, using a Wilcoxon rank-sum test, demonstrated a clear significant difference between the R (responders) and NR (nonresponders) groups. *P* values are shown in the figure. Download FIG S1, TIF file, 0.2 MB. Copyright © 2023 Olekhnovich et al. 2023 Olekhnovich et al. https://creativecommons.org/licenses/by/4.0/ This content is distributed under the terms of the Creative Commons Attribution 4.0 International license.

It is noteworthy that the data set used in the study of Frankel 2017 encompassed patients with different treatment regimens. Nevertheless, the Songbird approach identified the species that marked the differences between the R and NR, groups regardless of the immunotherapy protocol (Fig. S2).

10.1128/msystems.01023-22.2 FIG S2 Feature rankings and differentials generated by Songbird (A) as well as boxplots of the log ratios of the selected features (B), depending on the type of immunotherapy used in the Frankel 2017 dataset. The top 20 taxonomic features with both positive and negative differential values were selected for the analysis. The selected species that were subjected to the log ratios assessment are highlighted in blue and green colors, corresponding to the R and NR groups, respectively. The log-ratios statistical assessment, using a Wilcoxon rank-sum test, demonstrated a clear significant difference between the R (responders) and NR (nonresponders) groups but not between the groups that were subjected to different types of immunotherapy. *P* values are shown in the figure. The types of immunotherapy groups were: I, ipilimumab; N, nivolumab; IN, ipilimumab + nivolumab; P, pembrolizumab. Download FIG S2, TIF file, 0.1 MB. Copyright © 2023 Olekhnovich et al. 2023 Olekhnovich et al. https://creativecommons.org/licenses/by/4.0/ This content is distributed under the terms of the Creative Commons Attribution 4.0 International license.

The aim of analyzing the group 2 data sets was to investigate the impact of fecal microbiota transplantation (FMT) on RI. It should be noted that the FMT-related data are more complex than the group comparison data (Group 1), as they usually consist of donor metagenomes and time series of recipient metagenomes. Therefore, the identification of biomarkers in group 2 was carried out in two steps: (i) the detection of donor-derived microbes in the samples of recipients and (ii) the identification of donor-derived microbial species that colonized the recipient and were associated with the improvement of symptoms of melanoma after immunotherapy.

In the first step, the diversity of donor-derived microbes in the metagenomes of the recipients was determined using the RECAST approach. A total of at least 102 donor-derived bacterial species were identified in each recipient. The list of the top five bacteria that colonized the majority of recipients is: Eubacterium rectale, *F. prausnitzii*, *A. putredinis*, R. faecis, and Bacteroides uniformis (Fig. S3). According to variance analysis, the composition of donor-derived microbes was more dependent on the donor subject rather than on RMI in both of the FMT data sets (PERMANOVA, *R*^2^ = 0.20, *P*_adj_ < 0.05; *R*^2^ = 0.07, *P*_adj_ < 0.05, respectively, for Baruch 2021; *R*^2^ = 0.14, *P*_adj_ < 0.001; *R*^2^ = 0.09, *P*_adj_ < 0.001 for Davar 2021; Aitchison distance, 10,000 permutations).

**FIG 2:**
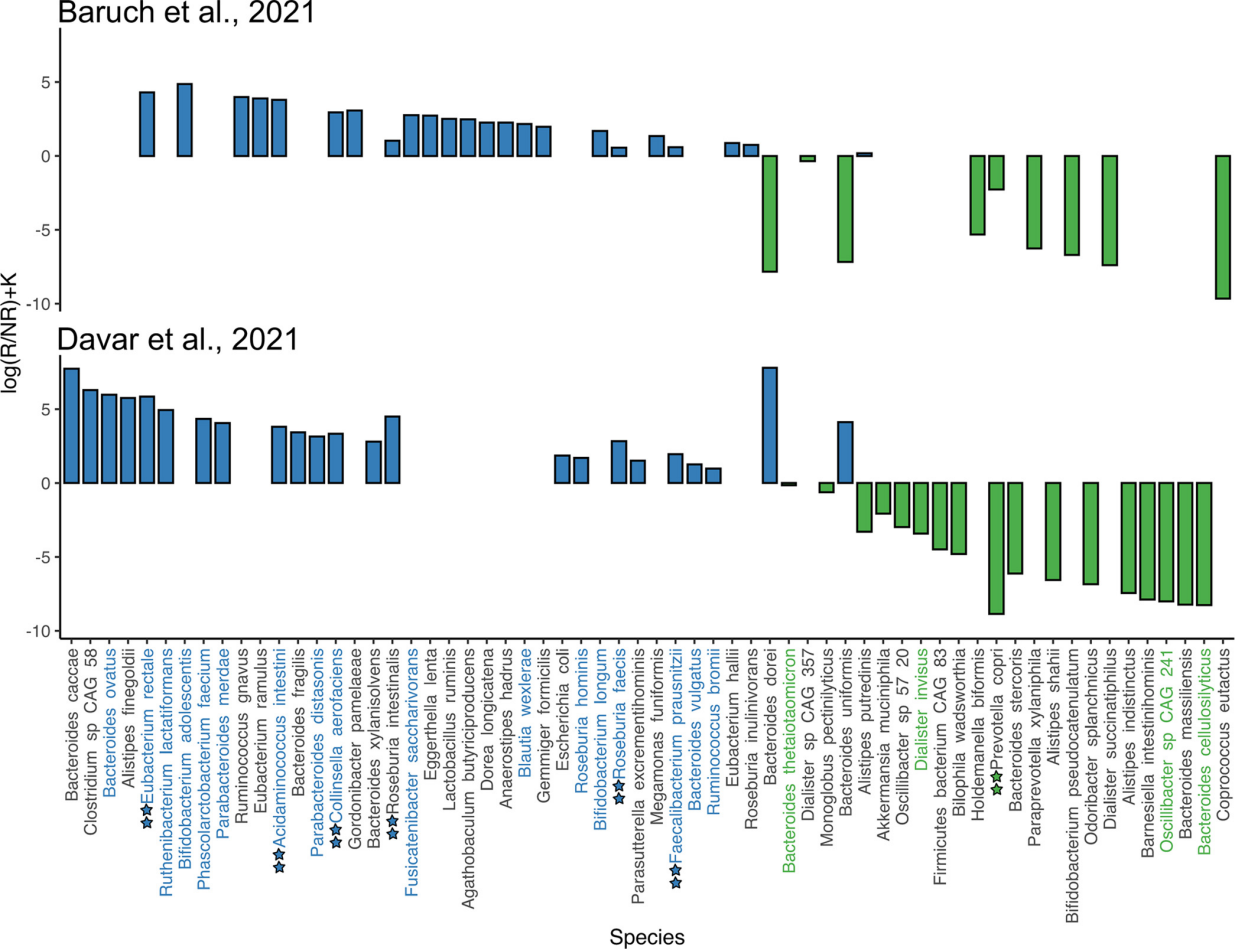
Donor-derived species rankings and differentials generated by Songbird. Microbial species are plotted on the *x* axis, and differentials describing the log-fold change in taxa associated with RI are plotted on the *y* axis. Positive coefficient levels correspond to an association with RMI and negative levels correspond to UMI. The colored stars designate species with positive or negative associations in both the Baruch 2021 and Davar 2021 data sets. Species that contain consistent biomarkers are highlighted in blue and green.

In the second step, Songbird was used to perform multinomial regression for the detection of donor-derived microbes that were associated with RMI. The log-ratio assessment of the Songbird differential rankings of donor-derived microbial profiles showed a clear, statistically significant difference between the R and NR groups (Fig. S4). As the analysis of the group 1 data sets showed, the predictors of RMI included E. reclate, Acidaminococcus intestini, Collinsella aerofaciens, Roseburia intestinalis, *R. faecis*, and *F. prausnitzii*. Only Prevotella copri was consistently associated with UMI (Fig. 2). Interestingly, E. rectale was a stronger predictor of RMI for the FMT-related data sets, in comparison to *F. prausnitzii*. Firmicutes appeared to be beneficial in both data sets (maintaining a difference in the species content), whereas Bacteroidetes and Actinobacteria demonstrated conflicting results in terms of their impacts on immunotherapy efficacy. For example, Bacteroides dorei and B. uniformis were strongly associated with RMI in the Davar 2021 data set but not in the Baruch 2021 data set. In contrast, *A. putredinis* was associated with a beneficial impact on immunotherapy in the Baruch 2021 data set but not in the Davar 2021 data set. Moreover, Actinobacteria emerged as a significant predictor of immunotherapy outcome only in the Baruch 2021 data set, whereas a major part of Bacteroidetes emerged as a significant predictor of RI only in the Davar 2021 data set. However, these differences may be due to technical biases that are linked to differences in DNA extraction procedures (18) but not with biological phenomena.

10.1128/msystems.01023-22.3 FIG S3 Donor-derived metagenomic reads per species identified in the post-FMT recipients’ metagenomes. Different colors showed different RI. The width of each dot corresponds to the number of metagenomic reads. The species that were added to the list of consistent biomarkers are highlighted in blue. Download FIG S3, TIF file, 1.7 MB. Copyright © 2023 Olekhnovich et al. 2023 Olekhnovich et al. https://creativecommons.org/licenses/by/4.0/ This content is distributed under the terms of the Creative Commons Attribution 4.0 International license.

The second stage of the analysis was to put together a list of consistent biomarkers following this scheme: (i) microbial species that were associated with RMI, according to the Songbird results in more than one data set, were added to the list; (ii) microbial species that were associated with UMI in at least one data set were excluded from the list of biomarkers, regardless of the number of data sets in which they were associated with RMI. The identified taxonomic biomarkers have been associated with RMI in other studies, which we mention in Table 1.

**TABLE 1:** Consistent taxonomic biomarkers associated with RMI, resulting from the analysis of the data sets for groups 1 and 2

Species (% of samples with nonzero relative abundance)	Phyla	Studies included in our analysis	Other studies
Faecalibacterium prausnitzii (94)	Firmicutes	Frankel 2017, Gopalakrishnan 2018, Matson 2018, Baruch 2021, Davar 2021	4, 5, 7–10, 13, 55, 56
Roseburia faecis (70)	Firmicutes	Gopalakrishnan 2018, Matson 2018, Baruch 2021, Davar 2021	14
Bacteroides ovatus (96)	Bacteroidetes	Gopalakrishnan 2018, Matson 2018, Davar 2021	57
Bacteroides vulgatus (99)	Bacteroidetes	Gopalakrishnan 2018, Matson 2018, Davar 2021	24, 58
Blautia wexlerae (80)	Firmicutes	Frankel 2017; Matson 2019, Baruch 2021	
Collinsella aerofaciens (79)	Actinobacteria	Matson 2018, Baruch 2021, Davar 2021	7
Eubacterium siraeum (52)	Firmicutes	Frankel 2017, Gopalakrishnan 2018, Matson 2018	56, 59
Roseburia hominis (71)	Firmicutes	Frankel 2017, Gopalakrishnan 2018, Davar 2021	14
Ruminococcus bromii (47)	Firmicutes	Frankel 2017; Gopalakrishnan 2018, Davar 2021	8, 60, 61
Acidaminococcus intestini (36)	Firmicutes	Baruch 2021, Davar 2021	
Bifidobacterium adolescentis (47)	Actinobacteria	Matson 2019, Baruch 2021	7, 61
Bifidobacterium longum (53)	Actinobacteria	Matson 2019, Baruch 2021	7, 19, 62, 63
Eubacterium rectale (87)	Firmicutes	Baruch 2021, Davar 2021	
Fusicatenibacter saccharivorans (79)	Firmicutes	Gopalakrishnan 2018, Baruch 2021	
Parabacteroides distasonis (96)	Bacteroidetes	Gopalakrishnan 2018, Davar 2021	24, 64
Parabacteroides merdae (89)	Bacteroidetes	Matson 2018, Davar 2021	7, 61
Phascolarctobacterium faecium (59)	Firmicutes	Gopalakrishnan 2018, Davar 2021	
Ruminococcus bicirculans (46)	Firmicutes	Frankel 2017, Gopalakrishnan 2018	
Ruthenibacterium lactatiformans (95)	Firmicutes	Matson 2018, Davar 2021	64

The resulting list of consistent taxonomic biomarkers that are associated with RMI included 19 bacterial species: 12 Firmicutes, 4 Bacteroides, and 3 Actinobacteria. Many major short-chain fatty acid producers, such as *F. prausnitzii*, *R. faecis*, *R. hominis*, *R. bromii*, and E. rectale, were included in the list. Interestingly, *F. prausnitzii* was identified to be a significant predictor of RMI in all of the data sets that were included in the analysis. In addition, we found nine studies in the scientific literature that also reported the beneficial role of *F. prausnitzii* in immunotherapy. All of the identified consistent taxonomic biomarkers coincided with the colonizers that were identified in the FMT data sets (Fig. S3). However, only 17 out of 19 (excluding *Ruminococcus bicirculans* and *E. siraeum*) were associated with RMI in the FMT-related data sets. Moreover, *E. reclate* and *A. intestini* were found to be predictive of RMI only in the FMT-related data sets. It should also be noted that *F. prausnitzii* and *Roseburia* spp. have been identified as cross-cohort biomarkers of a response to ICIs therapy in previously published meta-analyses (13, 14), which further confirms the roles of these bacteria in producing a positive response to ICIs therapy.

Consistent biomarkers associated with UMI were identified by applying the same methodology. The resulting list included 4 Firmicutes, 2 Bacteroides, 2 Actinobacteria, and 1 Proteobacterium; the species were Bacteroides thetaiotaomicron, Adlercreutzia equolifaciens, Asaccharobacter celatus, Bacteroides cellulosilyticus, Coprococcus comes, Dialister invisus, Firmicutes bacterium CAG 145, *Oscillibacter* sp CAG 241, and Proteobacteria bacterium CAG 139. However, only B. thetaiotaomicron was predictive of UMI in 4 data sets (Frankel 2017, Gopalakrishnan 2018, Matson 2018, Davar 2021). The rest of the bacteria from this list showed consistent results in only two data sets. Concurrently, in the group 2 data sets, there were no consistent biomarkers of UMI among the analyzed studies. Only *P. copri* displayed predictive power of UMI in both Baruch 2021 and Davar 2021. However, as it also displayed predictive power of RMI in Frankel 2017, it was excluded from the list.

### Validation of consistent taxonomic biomarkers using group comparison of metagenomics data sets.

To test the identified consistent taxonomic biomarkers, we applied group comparison to the metagenomic data sets that were published by Spencer 2021 and Lee 2022. There were a total of 322 metagenomes that were divided into 164 responders and 158 nonresponders. We compared the list of consistent biomarkers by using the Songbird differential values in Spencer 2021 and Lee 2022. Microbial species that exhibited contradictory results in at least one of the additional data sets were excluded from further analysis. As a result, only four bacterial species, namely, B. adolescentis, *F. prausnitzii*, E. rectale, and *P. merdae*, were found to be consistent biomarkers of RMI in additional data sets. One species, namely, *A. equolifaciens*, was found to be a biomarker of UMI. After further verification, *P. merdae* and *A. equolifaciens* were removed from this list, as their differential values at least in one of the additional data sets were <0.1, and they did not exhibit differences between the R and NR groups in the log-ratio analysis. Thus, from the list of early identified cross-study taxonomic biomarkers, only B. adolescentis, *F. prausnitzii*, and E. rectale were reproducible in the Spencer 2021 and Lee 2022 data sets. It is noteworthy that only *F. prausnitzii* was found to be a predictor of RMI in all seven of the data sets that were included in the analysis and that E. rectale and B. adolescentis were consistent in four of the data sets. Next, we carried out a log-ratio assessment of B. adolescentis, *F. prausnitzii*, and E. rectale across metagenomes in additional data sets. Since representative biomarkers of UMI were not identified, only taxa with differential values of <−0.5 were selected as a set of denominators.

**FIG 3:**
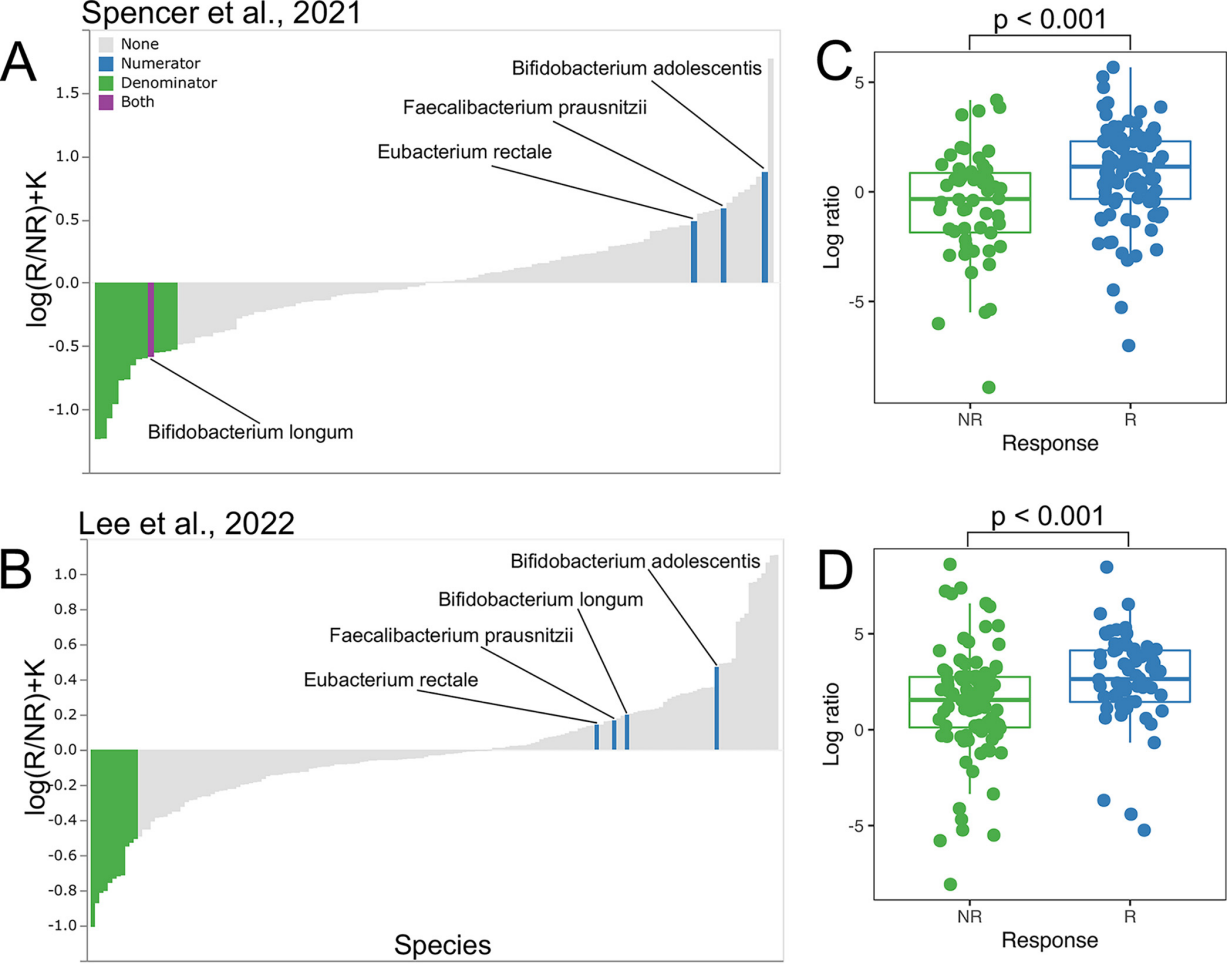
Validation of the consistent taxonomic biomarkers associated with RMI or UMI using additional metagenomic data sets. (A and B) Feature rankings and differentials generated by Songbird. Microbial species are plotted on the *x* axis, and differentials describing the log-fold change in features with regard to RI are plotted on the *y* axis. The validated consistent taxonomic biomarkers of RMI are highlighted in blue. Taxonomic features with differential values of <−0.5 that were selected as a set of denominators are highlighted in green. (C and D) Boxplots of the log ratios of the selected features are highlighted in color in panels A and B. The log-ratios statistical assessment, which was performed using the Wilcoxon rank-sum test, shows a clear significant difference between the R (responders) and NR (nonresponders) groups. *P* values are shown in the figure. The plots in panels A and C correspond to the Spencer 2018 data set, and the plots in panels B and D correspond to the Lee 2022 data set.

Today, we know that many species of bifidobacteria, by stimulating the immune system, can improve the response to ICIs therapy (19, 20). However, in our study, only B. adolescentis displayed strong predictive power of a RMI, whereas another bifidobacteria species was associated with UMI. To demonstrate these effects, we added B. longum to the log-ratio assessment, and it was found to be a reproducible predictor of RMI in the group 1 and 2 data sets but not in the additional data sets. As a result, the log-ratio assessment of the selected taxa across metagenomes showed a clear, statistically significant difference between the R and NR groups in both the Spencer 2021 and Lee 2022 data sets. The results are presented in Fig. 3. The Songbird differential values across the identified taxonomic biomarkers and data sets are presented in Table S2.

10.1128/msystems.01023-22.6 TABLE S2 Consistent taxonomic biomarkers associated with immunotherapy outcome with differential coefficients. Download Table S2, XLSX file, 0.01 MB. Copyright © 2023 Olekhnovich et al. 2023 Olekhnovich et al. https://creativecommons.org/licenses/by/4.0/ This content is distributed under the terms of the Creative Commons Attribution 4.0 International license.

### Discovery of consistent functional biomarkers associated with RI.

The discovery of consistent functional biomarkers that are associated with RMI was achieved by employing the same approach as that used in the taxonomic analysis. The top 200 functional features displaying positive or negative differential values were selected for further analysis. The log-ratio assessment of the selected KEGG orthology groups (KOG) of the analyzed metagenomes or donor-derived microbial profiles for the additional data sets demonstrated a clear, statistically significant difference between the R and NR groups (Wilcoxon rank-sum test, *P* < 0.001). A total of 140 KOGs that were associated with RMI in more than one data set were identified as a result of analyzing the group 1 and 2 data sets, whereas 57 KOGs were associated with poor RMI. After the validation of the results using the Spencer 2021 and Lee 2022 data sets, 101 KOGs were associated with RMI, and 20 KOGs were associated with poor RMI (Table S3). Among the list of consistent functional biomarkers of RMI were KOGs that are involved in polysaccharide metabolism (K16148, K16147, K01210, K01218, K01136) as well as in peptidoglycan (K07284, K05364, K12554) and fatty acid biosynthesis (K11533, K11263). It should be noted that the gluABCD glutamate uptake system (K10005, K10006, K10007, K10008) and the phosphotransferase (PTS) encoded by the gfrABCD operon (K19506, K19507, K19508, K19509), which leads to the utilization of Maillard reaction products (MRPs), such as fructoselysine/glucoselysine, are also predictors of RMI. Among other significant predictors of RMI were gene groups that are involved in the biosynthesis of cofactors, such as cobalamin (K13542) and ubiquinone (K03688). Moreover, the KOGs corresponding to sporulation (K18955, K06383, K06297, K06313, K06330, K06413) and motility/secretion systems (K02653, K02398, K02417, K02278) were also associated with RMI. Concurrently, among other functional biomarkers of UMI were KOGs derived from pathogenic bacteria, such as KOGs that are involved in aerobactin biosynthesis (K03894, K03895, K03896, K03897).

10.1128/msystems.01023-22.7 TABLE S3 Consistent functional biomarkers associated with immunotherapy outcome with differential coefficients. Download Table S3, XLSX file, 0.02 MB. Copyright © 2023 Olekhnovich et al. 2023 Olekhnovich et al. https://creativecommons.org/licenses/by/4.0/ This content is distributed under the terms of the Creative Commons Attribution 4.0 International license.

### Identification of the relation between taxonomic and functional biomarkers of RI.

**FIG 4:**
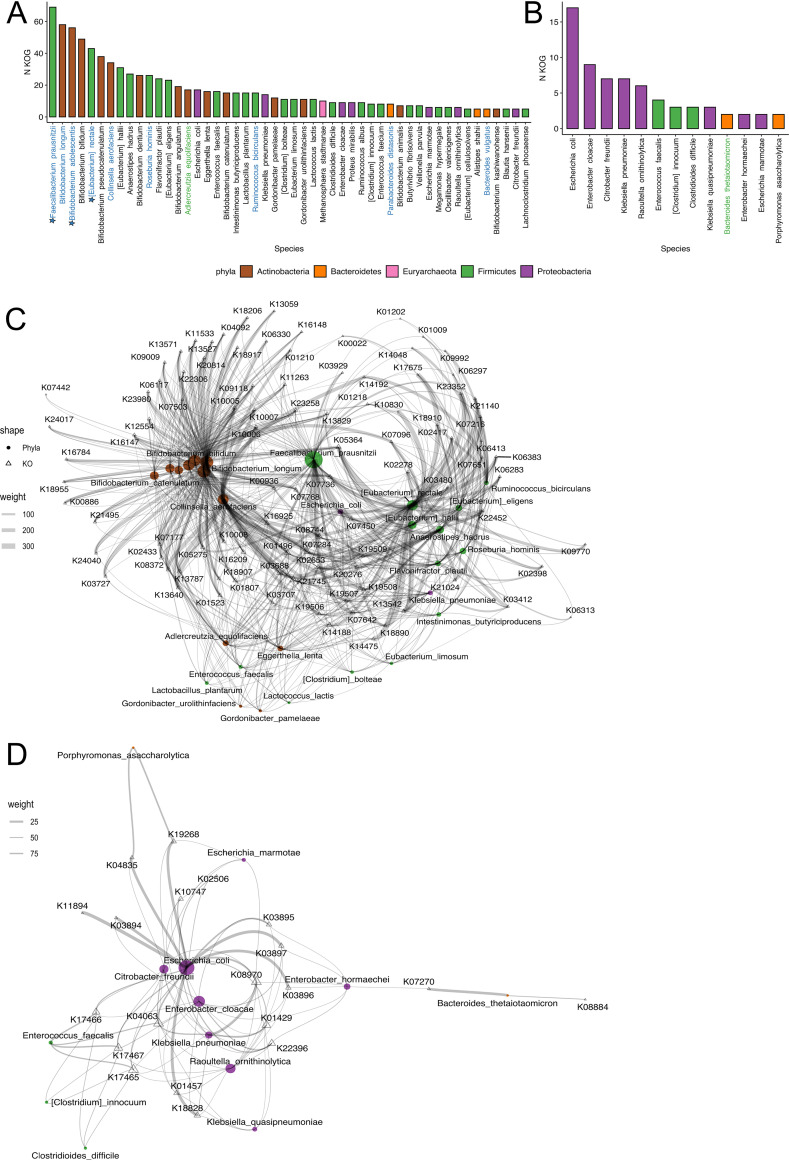
Taxonomic affiliation of consistent functional biomarkers of RI. (A and B) The number of KOGs linked to each bacterial species, as identified by Kraken 2. The bacterial species are plotted on the *x* axis, and the numbers of KOGs are plotted on the *y* axis. The color bars indicate the phyla of the bacteria. The species that have been selected as consistent taxonomic biomarkers in the group 1 and group 2 data sets are highlighted in blue and green. The taxonomic biomarkers associated with RMI and validated on the Spencer 2021 and Lee 2022 data sets are marked by blue stars. (C and D) The networks show taxonomic affiliations belonging to validated positive functional biomarkers. Triangles correspond to bacterial phyla, and circles correspond to KOGs. The widths of the circles correspond to the numbers of related KOGs. The plots in panels A and C correspond to the validated consistent functional biomarkers that are associated with RMI, whereas the plots in panels B and D correspond to UMI.

Using the MetaCharchant and Kraken2 tools, we studied the relationship between the taxonomic results and the consistent functional biomarkers described above (Table S4). According to the results presented in Fig. 4A, 45 Firmicutes, 18 Actinobacteria, 17 Proteobacteria, 11 Bacteroidetes, 3 Euryarchaeota, 1 Synergistetes, and 1 Verrucomicrobia bacteria were linked to functional biomarkers that are associated with RMI. It is noteworthy that 9 out of 19 phyla (approximately 47%) have been included in the group 1 and 2 data sets taxonomic biomarkers that are associated with RMI, which are presented in Table 1. According to a variance analysis, different bacterial phyla were linked to different biomarkers (PERMANOVA, *R*^2^ = 0.12, *P* < 0.001; Bray-Curtis dissimilarity, 10,000 permutations). The number of KOGs associated with RMI was highest in the species belonging to Actinobacteria (Wilcoxon rank-sum test, *P*_adj_ < 0.05). Firmicutes, Bacteroidetes, and Proteobacteria were least linked to the selected gene groups, even though the results were not statistically significant (Wilcoxon rank-sum test, *P*_adj_ > 0.05). The top five bacteria that contained KOGs that were most strongly linked to RMI were: *F. prausnitzii*, B. longum, B. adolescentis, Bifidobacterium bifidum, and E. rectale. It is noteworthy that this list is partially similar to the list of consistent taxonomic biomarkers that resulted from the analysis of the Spencer 2021 and Lee 2022 data sets (*F. prausnitzii*, B. adolescentis, and E. rectale) (Fig. 3). Interestingly, a combination of *F. prausnitzii*-linked KOGs and any of the bifidobacteria B. longum, B. adolescentis and B. bifidum was seen in approximately 85% of the cases of consistent functional biomarkers of RMI.

10.1128/msystems.01023-22.8 TABLE S4 Links between functional biomarkers and bacterial species. Download Table S4, XLSX file, 0.8 MB. Copyright © 2023 Olekhnovich et al. 2023 Olekhnovich et al. https://creativecommons.org/licenses/by/4.0/ This content is distributed under the terms of the Creative Commons Attribution 4.0 International license.

In contrast, 8 Proteobacteria, 3 Firmicutes, and 2 Bacteroidetes bacteria were associated with functional biomarkers of UMI. The top five bacterial species that contained the highest numbers of functions associated with biomarkers of UMI were the opportunistic and potential opportunistic species Escherichia coli, Enterobacter cloacae, Citrobacter freundii, Klebsiella pneumoniae, and Raoultella ornithinolytica (Fig. 4B). All of the identified aerobactin biosynthesis KOGs belonged to E. coli and E. cloacae. It is noteworthy that we identified bacterial species, including Escherichia coli, E. cloacae, B. thetaiotaomicron, and others, that are linked to functional biomarkers of both RMI and UMI.

We also carried out a network analysis of the relationship between the taxonomic results and the consistent functional biomarkers. The bacterial phyla that differed in their content of functional biomarkers of RMI were also identified (Fig. 4C). Interestingly, *F. prausnitzii* occupied an intermediate position between the Firmicutes and Actinobacteria clusters, and it was linked to the specific KOGs of both of the bacterial phyla. The functional biomarkers of UMI were predominantly linked to Proteobacteria and did not form distinct clusters (Fig. 4D).

## DISCUSSION

The international scientific community is being actively engaged in studying the influence of the human intestinal microbiota on the efficacy of cancer immunotherapy. However, despite the large number of published studies, researchers still cannot find reliable and consistent gut microbial determinants of RMI. Thus, the development and validation of new tools of metagenomic analysis and reanalysis of the published data may help us reveal new links between the composition of the gut microbiome and the efficacy of immunotherapy. In this study, we focused on melanoma-related metagenomic data due to its abundance, in comparison with data related to other tumor types. Using the Songbird tool for compositional data analysis (21), we analyzed the gut metagenomes from seven published studies and identified three bacterial species as cross-study taxonomic biomarkers that are associated with RI: *F. prausnitzii*, B. adolescentis, and E. rectale. Nevertheless, only *F. prausnitzii* was consistent across the data sets that were included in the analysis. It is noteworthy that the identified biomarkers were congruent with the results of other published studies (Table 1). Moreover, despite the fact that the Frankel 2017 and Lee 2022 data sets included patients who were exposed to different treatment regimens (anti-PD1, anti-CTLA4, or a combination of both), the identified biomarkers still described the difference between the R and NR patient groups well. Many have suggested that *F. prausnitzii*, E. rectale, and bifidobacteria exhibit immunomodulatory potential in relation to both healthy people and COVID-19 patients (22, 23). We assume that there are universal stool metagenomic biomarkers that are reflective of the state of the immune system. Nonetheless, extensive clinical studies are needed to confirm these hypotheses.

Furthermore, we identified consistent functional biomarkers of RMI that are related to polysaccharide metabolism, peptidoglycan and fatty acid biosynthesis, the gluABCD glutamate uptake system, fructoselysine/glucoselysine utilization by gfrABCD PTS, as well as groups of genes that are involved in the biosynthesis of cofactors, such as cobalamin and ubiquinone. Interestingly, the groups of genes related to sporulation and motility/secretion systems were predictive of RMI. Today, we know that the gut microbial community promotes a variety of digestive metabolic functions, some of which may be associated with the improved functioning of the immune system and RMI. The identified biomarkers are corroborated by the results of other published findings regarding the molecular mechanisms involved in RI. For example, the increased intake of plant polysaccharides improves RMI (10, 24). Moreover, *F. prausnitzii*, E. rectale, and B. adolescentis, which were pinpointed as taxonomic biomarkers, are major producers of butyrate and other short-chain fatty acids. Gut microbiome-derived SCFAs are necessary for fine-tuning the body’s metabolism and cytotoxic immunity, both of which improve the efficacy of immunotherapy (25–27). Moreover, gene groups that are involved in sporulation and motility can be characterized as pattern recognition receptor (PRR) ligands, and they are involved in the modulation of host immunity and in the efficacy of immunotherapy (20, 28–31). Another finding of this study that may be relevant to the modulation of RMI by gut microbes is that the gut bacteria may affect food safety by tweaking the generation of Maillard reaction products (32). We determined that the gfrABCD PTS system, which is used by bacteria as a fructoselysine/glucosolysine disposal system, can be utilized for the prediction of immunotherapy success. This discovery fits well in our paradigm, as fructoselysine may be used by intestinal bacteria as an additional substrate for butyrate production (33), which thereby boosts the probiotic component of the microbiome and increases the body’s receptivity of cancer immunotherapy.

Using our previously developed computational pipeline (16), we ranked the bacterial species by the numbers of consistent functional biomarkers contained in them. In total, 96 bacterial species were linked, to some extent, to KOG predictors of RMI. Based on these results, we hypothesized that the irregularities between previous studies might be explained by the fact that beneficial functions are distributed among different bacterial species. Only *F. prausnitzii* contained approximately 70% of the identified functions that are beneficial to immunotherapy success. Moreover, multiple studies demonstrated the crucial role of *F. prausnitzii* in determining the body’s RI (Table 1). Thus, *F. prausnitzii* appears to be a key determinant of immunotherapy success. Interestingly, the combination of *F. prausnitzii*-linked KOGs with any of the bifidobacteria B. longum, B. adolescentis, or B. bifidum covered approximately 85% of the identified consistent biomarkers of successful immunotherapy. It is possible that rational design of probiotics must take into account the symbiotic bonds between beneficial bacteria, as they can lead to synergetic beneficial effects, overall. Studies have already shown that the combination of *F. prausnitzii* and bifidobacteria improves butyrate production, growth, and gut colonization (34, 35). Although there is no doubt that bifidobacteria exert a wide range of positive properties related to human immunity, according to our results, only B. adolescentis was a predictor of successful immunotherapy. Concurrently, despite the large number of immunotherapy-related beneficial KOGs that were harbored by B. longum, the results of the validation of the taxonomic biomarkers for this species were not reproducible. Moreover, B. bifidum was not identified to be a consistent taxonomic predictor, despite being associated with a large number of beneficial KOGs. We conjecture that these results might be explained by the fact that the beneficial properties exhibited by bifidobacteria toward immunotherapy are strain-specific (20) or can be manifested via interactions with other intestinal microorganisms.

In addition to searching for biomarkers of RMI, we set out to establish which distinctive features, other than a low abundance of beneficial microbes, are unique to NRs. However, we did not find any representative taxonomic biomarkers in NRs. Interestingly, the “Anna Karenina principle” seems to be applicable to animal microbiomes in the sense that, unlike balanced microbiomes, which seem to have common features, dysbiotic microbiomes have little to none, paralleling Leo Tolstoy’s dictum that “All happy families look alike; each unhappy family is unhappy in its own way.” (36). Nevertheless, we identified functional biomarkers of UMI, such as the KOGs that are involved in aerobactin biosynthesis. As reported by many studies, the production of aerobactin is a virulence factor that has been found in intestinal pathogens. We identified some links between aerobactin biosynthesis and both E. coli and E. cloacae. It is noteworthy that E. coli has also been linked to a large number of functional biomarkers that are characteristic of RMI. We conjecture that E. coli strains can be both beneficial or opportunistic in regard to melanoma immunotherapy. In addition, we could not conclude which group B. thetaiotaomicron belongs to, as it did not display any distinctive pattern between the compared data sets. This is congruent with the literature, which states that the role of B. thetaiotaomicron in modulating immunotherapy outcome is ambiguous. On the one hand, the administration of B. thetaiotaomicron improved RMI in mice (28). On the other hand, B. thetaiotaomicron was suggested as a determinant of UMI (8). B. thetaiotaomicron has also been blamed for exacerbating enteric infections (37, 38). Thus, it is feasible that ascribing a concrete status to B. thetaiotaomicron depends to a large extent on the presence of pathogenic bacteria in the metagenome.

The impact of fecal microbiota transplantation on RI was strongly dependent on the donor’s microbiome. However, the most beneficial donated samples did not work alike for all of the patients. In other words, the FMT immunomodulatory success depends on both the characteristics of the donor microbiome and the recipient’s intestinal environment (or other unknown factors). Moreover, species that have been linked to successful therapy have also colonized NRs. It is possible that the engraftment of a broad array of donor-derived species is a side effect of FMT (39). At the same time, unique, hidden aspects of the donor microbiome, such as molecular structures that are secreted by specific strains or certain bacteriophages, can account for RMI (20, 40). Also, other factors, such as the microbial load (which cannot be assessed using metagenomic data) in the donor stool samples and/or in the recipient’s mucosa (41), may be a strong determinant of the success of FMT. Interestingly, the most efficient microbial colonizers, namely, *F. prausnitzii* and E. rectale, were also associated the most with beneficial KOGs, and they were included in the list of validated consistent taxonomic biomarkers. One possible explanation is that the adaptive evolutionary processes that led to the selection of the most beneficial species for the host also led to the selection of the most competitive microbial symbionts.

### Conclusions.

Today, it is widely known that the gut microbiome exerts many positive effects on the functioning of the host organism, ranging from metabolic functions to the influence of microbial-derived molecular structures on cell-specific receptors. Since metagenomic sequencing is not suitable for quantifying the fecal microbial load, we can assume that the general microbiome signature detected by human cells is degraded in nonresponders, in comparison to responders, due to infection or some other reason, and that the identified biomarkers are indicators of the microbiome structure degradation. Moreover, the specific characteristics of the Rs’ intestinal communities, which are transferable from one person to another, may determine the effectiveness of immunotherapy. To put it briefly, the true biological nature of the observed phenomena has not yet been established. Nevertheless, some useful conclusions can be drawn. For example, the inclusion of digestive fibers and probiotic strains of bifidobacteria into treatment regimens will definitely improve the chances of successful immunotherapy. However, the question of which, among commercial probiotics, is the most effective remains open. In addition, it is likely that taking steps toward enhancing intestinal infection control will also improve the efficacy of ICIs.

## MATERIALS AND METHODS

### Metagenomic data.

Sequencing data from samples of gut metagenomes of melanoma patients who displayed different responses to ICIs were collected before any interventions from three published studies (5, 7, 8). These data were used to analyze the associations between taxonomic or functional metagenomic profiles and the responsiveness of patients to immunotherapy. Additional data that showed the impact of FMT on immunotherapy (11, 12) were used to validate the identified biomarkers. To validate the selected biomarkers, two additional metagenomic data sets were used (10, 14). In total, 680 metagenomic stool samples were used in the study, of which 374 metagenomes were from melanoma patients with RMI (responders group, R) and 306 metagenomes were from melanoma patients who were unresponsive to immunotherapy (nonresponders group, NR). The characteristics of the metagenomic data sets that were used in the study are presented in Table 2.

**TABLE 2:** Characteristics of the metagenomic data sets that were used in the study

Dataset	Immunotherapy type	Individuals/Samples	Responders/Nonresponders	Reads per metagenome (mean ± SD), mln	Sequencing platform (read length, bp)
Frankel 2017	anti-PD1/anti-CTLA4	39/39	19/20	36.8 ± 10.6	Illumina (100)
Gopalakrishnan 2018	anti-PD1	25/25	14/11	15.9 ± 4.0	Illumina (100)
Matson 2018	anti-PD1	38/38	14/24	39.7 ± 16.9	Illumina (150)
Baruch 2021	anti-PD1	10/42	3/7	15.8 ± 5.5	Illumina (150)
Davar 2021	anti-PD1	15/214	3/12	11.1 ± 3.8	Illumina (150)
Spencer 2021	anti-PD1	158/158	100/58	12.5 ± 7.8	Illumina (100 to 150)
Lee 2022	anti-PD1/anti-CTLA4	164/164	100/64	24.7 ± 18.3	Illumina (150)

### Data analysis strategy.

Raw metagenomic data were downloaded from the NCBI/EBI public data repositories, using the SRA-Toolkit’s fastq-dump tool (42). The assessment of read quality and the filtering out of poor quality reads were carried out using the FastQC software package (https://github.com/s-andrews/FastQC). Technical sequences and bases with a quality of lower than a 30 Phred score were trimmed using Trimmomatic (43). Human sequences were removed from the metagenomes using bbmap (44) and the GRCh37 human genome version. The described preprocessing computational steps that were applied to the metagenomics reads were implemented in the Assnake metagenomics pipeline (https://github.com/ASSNAKE). The donor-derived reads of post-FMT stool metagenomes in FMT-related data sets were identified using the RECAST algorithm (17). The taxonomic and functional profiles of the processed stool metagenomes or the donor-derived metagenomic reads were obtained using the MetaPhlAn3 (45) and HUMAnN2 approaches (46) along with the KEGG database (47).

The identification of consistent taxonomic and functional biomarkers that are associated with RMI was performed in two steps. For the first step, using the taxonomic and functional profiles of stool metagenomes or donor-derived reads (for the FMT-related data), microbial species with increased or decreased abundance were identified in patients who were responsive to immunotherapy using the Songbird (21) and Qurro (48) tools that are implemented in the QIIME2 framework (49). The second step was the creation of a list of consistent biomarkers, which was done by following this methodology: (i) microbial species that were associated with RMI in more than one data set were added to the list; (ii) microbial species that were associated with UMI in at least one data set were excluded from the list of biomarkers, regardless of the numbers of data sets in which they were associated with a positive outcome. The specific UMI biomarkers were identified in a similar way.

The search for links between taxonomic and functional biomarkers was carried out as follows. The identification of taxonomic affiliations and the reconstruction of the metagenomic context of the functional biomarkers was performed using the MetaCherchant tool (16). Unitigs with a length of >1,000 bp were selected for subsequent taxonomic annotation using Kraken 2 (50) and a precomputed database (https://lomanlab.github.io/mockcommunity/mc_databases.html). The resulting data were processed using the ggraph package for GNU/R (51).

Additional statistical analyses and visualizations were carried out using the vegan package (52), ggplot2 library, and standard statistical techniques that are implemented in GNU/R. The PERMANOVA (adonis function of the vegan package), Bray-Curtis dissimilarity (52), and Aitchison distance (53, 54) were used for measuring the distances and dissimilarities in the comparisons of the taxonomy profiles of the read categories and functional biomarkers content. The Benjamini-Hochberg procedure was used to correct for multiple hypothesis testing.

### Data availability.

In this study, we used data from open sources, which are available at the NCBI and EBI Sequence Read Archives under the BioProject accession numbers PRJNA397906, PRJEB22893, PRJNA399742, PRJNA678737, PRJNA672867, PRJNA770295, and PRJEB43119. All of the results of the project are presented in the article text and in the additional materials.

10.1128/msystems.01023-22.4 FIG S4 Feature rankings and differentials generated by Songbird (A and C) as well as boxplots of the log ratios of the selected features (B and D). The selected species that were subjected to the log ratios assessment are highlighted in blue and green colors, corresponding to the R and NR groups, respectively. The log-ratio statistical assessment, using a Wilcoxon rank-sum test, showed a clear significant difference the between R (responders) and NR (nonresponders) groups. *P* values are shown in the figure. Download FIG S4, TIF file, 0.1 MB. Copyright © 2023 Olekhnovich et al. 2023 Olekhnovich et al. https://creativecommons.org/licenses/by/4.0/ This content is distributed under the terms of the Creative Commons Attribution 4.0 International license.

## References

[1] Borghaei H, Paz-Ares L, Horn L, Spigel DR, Steins M, Ready NE, Chow LQ, Vokes EE, Felip E, Holgado E, Barlesi F, Kohlhäufl M, Arrieta O, Burgio MA, Fayette J, Lena H, Poddubskaya E, Gerber DE, Gettinger SN, Rudin CM, Rizvi N, Crinò L, Blumenschein GR, Antonia SJ, Dorange C, Harbison CT, Graf Finckenstein F, Brahmer JR. 2015. Nivolumab versus docetaxel in advanced nonsquamous non–small-cell lung cancer. N Engl J Med 373:1627–1639. doi:10.1056/NEJMoa1507643. 26412456 PMC5705936

[2] Robert C, Schachter J, Long GV, Arance A, Grob JJ, Mortier L, Daud A, Carlino MS, McNeil C, Lotem M, Larkin J, Lorigan P, Neyns B, Blank CU, Hamid O, Mateus C, Shapira-Frommer R, Kosh M, Zhou H, Ibrahim N, Ebbinghaus S, Ribas A. KEYNOTE-006 investigators. 2015. Pembrolizumab versus ipilimumab in advanced melanoma. N Engl J Med 372:2521–2532. doi:10.1056/NEJMoa1503093. 25891173

[3] Horvat TZ, Adel NG, Dang T-O, Momtaz P, Postow MA, Callahan MK, Carvajal RD, Dickson MA, D'Angelo SP, Woo KM, Panageas KS, Wolchok JD, Chapman PB. 2015. Immune-related adverse events, need for systemic immunosuppression, and effects on survival and time to treatment failure in patients with melanoma treated with ipilimumab at Memorial Sloan Kettering Cancer Center. JCO 33:3193–3198. doi:10.1200/JCO.2015.60.8448. PMC5087335 26282644

[4] Chaput N, Lepage P, Coutzac C, Soularue E, Le Roux K, Monot C, Boselli L, Routier E, Cassard L, Collins M, Vaysse T, Marthey L, Eggermont A, Asvatourian V, Lanoy E, Mateus C, Robert C, Carbonnel F. 2017. Baseline gut microbiota predicts clinical response and colitis in metastatic melanoma patients treated with ipilimumab. Ann Oncol 28:1368–1379. doi:10.1093/annonc/mdx108. 28368458

[5] Frankel AE, Coughlin LA, Kim J, Froehlich TW, Xie Y, Frenkel EP, Koh AY. 2017. Metagenomic shotgun sequencing and unbiased metabolomic profiling identify specific human gut microbiota and metabolites associated with immune checkpoint therapy efficacy in melanoma patients. Neoplasia 19:848–855. doi:10.1016/j.neo.2017.08.004. 28923537 PMC5602478

[6] Routy B, Le Chatelier E, Derosa L, Duong CPM, Alou MT, Daillère R, Fluckiger A, Messaoudene M, Rauber C, Roberti MP, Fidelle M, Flament C, Poirier-Colame V, Opolon P, Klein C, Iribarren K, Mondragón L, Jacquelot N, Qu B, Ferrere G, Clémenson C, Mezquita L, Masip JR, Naltet C, Brosseau S, Kaderbhai C, Richard C, Rizvi H, Levenez F, Galleron N, Quinquis B, Pons N, Ryffel B, Minard-Colin V, Gonin P, Soria J-C, Deutsch E, Loriot Y, Ghiringhelli F, Zalcman G, Goldwasser F, Escudier B, Hellmann MD, Eggermont A, Raoult D, Albiges L, Kroemer G, Zitvogel L. 2018. Gut microbiome influences efficacy of PD-1–based immunotherapy against epithelial tumors. Science 359:91–97. doi:10.1126/science.aan3706. 29097494

[7] Matson V, Fessler J, Bao R, Chongsuwat T, Zha Y, Alegre ML, Luke JJ, Gajewski TF. 2018. The commensal microbiome is associated with anti–PD-1 efficacy in metastatic melanoma patients. Science 359:104–108. doi:10.1126/science.aao3290. 29302014 PMC6707353

[8] Gopalakrishnan V, Spencer CN, Nezi L, Reuben A, Andrews MC, Karpinets TV, Prieto PA, Vicente D, Hoffman K, Wei SC, Cogdill AP, Zhao L, Hudgens CW, Hutchinson DS, Manzo T, Petaccia de Macedo M, Cotechini T, Kumar T, Chen WS, Reddy SM, Szczepaniak Sloane R, Galloway-Pena J, Jiang H, Chen PL, Shpall EJ, Rezvani K, Alousi AM, Chemaly RF, Shelburne S, Vence LM, Okhuysen PC, Jensen VB, Swennes AG, McAllister F, Marcelo Riquelme Sanchez E, Zhang Y, Le Chatelier E, Zitvogel L, Pons N, Austin-Breneman JL, Haydu LE, Burton EM, Gardner JM, Sirmans E, Hu J, Lazar AJ, Tsujikawa T, Diab A, Tawbi H, Glitza IC, et al. 2018. Gut microbiome modulates response to anti–PD-1 immunotherapy in melanoma patients. Science 359:97–103. doi:10.1126/science.aan4236. 29097493 PMC5827966

[9] Peters BA, Wilson M, Moran U, Pavlick A, Izsak A, Wechter T, Weber JS, Osman I, Ahn J. 2019. Relating the gut metagenome and metatranscriptome to immunotherapy responses in melanoma patients. Genome medicine 11:1–14. doi:10.1186/s13073-019-0672-4. 31597568 PMC6785875

[10] Spencer CN, McQuade JL, Gopalakrishnan V, McCulloch JA, Vetizou M, Cogdill AP, Khan MAW, Zhang X, White MG, Peterson CB, Wong MC, Morad G, Rodgers T, Badger JH, Helmink BA, Andrews MC, Rodrigues RR, Morgun A, Kim YS, Roszik J, Hoffman KL, Zheng J, Zhou Y, Medik YB, Kahn LM, Johnson S, Hudgens CW, Wani K, Gaudreau P-O, Harris AL, Jamal MA, Baruch EN, Perez-Guijarro E, Day C-P, Merlino G, Pazdrak B, Lochmann BS, Szczepaniak-Sloane RA, Arora R, Anderson J, Zobniw CM, Posada E, Sirmans E, Simon J, Haydu LE, Burton EM, Wang L, Dang M, Clise-Dwyer K, Schneider S, et al. 2021. Dietary fiber and probiotics influence the gut microbiome and melanoma immunotherapy response. Science 374:1632–1640. doi:10.1126/science.aaz7015. 34941392 PMC8970537

[11] Baruch EN, Youngster I, Ben-Betzalel G, Ortenberg R, Lahat A, Katz L, Adler K, Dick-Necula D, Raskin S, Bloch N, Rotin D, Anafi L, Avivi C, Melnichenko J, Steinberg-Silman Y, Mamtani R, Harati H, Asher N, Shapira-Frommer R, Brosh-Nissimov T, Eshet Y, Ben-Simon S, Ziv O, Khan MAW, Amit M, Ajami NJ, Barshack I, Schachter J, Wargo JA, Koren O, Markel G, Boursi B. 2021. Fecal microbiota transplant promotes response in immunotherapy-refractory melanoma patients. Science 371:602–609. doi:10.1126/science.abb5920. 33303685

[12] Davar D, Dzutsev AK, McCulloch JA, Rodrigues RR, Chauvin J-M, Morrison RM, Deblasio RN, Menna C, Ding Q, Pagliano O, Zidi B, Zhang S, Badger JH, Vetizou M, Cole AM, Fernandes MR, Prescott S, Costa RGF, Balaji AK, Morgun A, Vujkovic-Cvijin I, Wang H, Borhani AA, Schwartz MB, Dubner HM, Ernst SJ, Rose A, Najjar YG, Belkaid Y, Kirkwood JM, Trinchieri G, Zarour HM. 2021. Fecal microbiota transplant overcomes resistance to anti–PD-1 therapy in melanoma patients. Science 371:595–602. doi:10.1126/science.abf3363. 33542131 PMC8097968

[13] Limeta A, Ji B, Levin M, Gatto F, Nielsen J. 2020. Meta-analysis of the gut microbiota in predicting response to cancer immunotherapy in metastatic melanoma. JCI insight 5. doi:10.1172/jci.insight.140940. PMC7714408 33268597

[14] Lee KA, Thomas AM, Bolte LA, Björk JR, de Ruijter LK, Armanini F, Asnicar F, Blanco-Miguez A, Board R, Calbet-Llopart N, et al. 2022. Cross-cohort gut microbiome associations with immune checkpoint inhibitor response in advanced melanoma. Nat Med 1–10. 35228751 10.1038/s41591-022-01695-5 PMC8938272

[15] Gloor GB, Macklaim JM, Pawlowsky-Glahn V, Egozcue JJ. 2017. Microbiome datasets are compositional: and this is not optional. Front Microbiol 8:2224. doi:10.3389/fmicb.2017.02224. 29187837 PMC5695134

[16] Olekhnovich EI, Vasilyev AT, Ulyantsev VI, Kostryukova ES, Tyakht AV. 2018. MetaCherchant: analyzing genomic context of antibiotic resistance genes in gut microbiota. Bioinformatics 34:434–444. doi:10.1093/bioinformatics/btx681. 29092015

[17] Olekhnovich EI, Ivanov AB, Ulyantsev VI, Ilina EN. 2021. Separation of donor and recipient microbial diversity allows determination of taxonomic and functional features of gut microbiota restructuring following fecal transplantation. mSystems 6:e00811–21. doi:10.1128/mSystems.00811-21. 34402648 PMC8407411

[18] Yang F, Sun J, Luo H, Ren H, Zhou H, Lin Y, Han M, Chen B, Liao H, Brix S, Li J, Yang H, Kristiansen K, Zhong H. 2020. Assessment of fecal DNA extraction protocols for metagenomic studies. GigaScience 9:giaa071. doi:10.1093/gigascience/giaa071. 32657325 PMC7355182

[19] Sivan A, Corrales L, Hubert N, Williams JB, Aquino-Michaels K, Earley ZM, Benyamin FW, Lei YM, Jabri B, Alegre M-L, Chang EB, Gajewski TF. 2015. Commensal Bifidobacterium promotes antitumor immunity and facilitates anti–PD-L1 efficacy. Science 350:1084–1089. doi:10.1126/science.aac4255. 26541606 PMC4873287

[20] Lee S-H, Cho S-Y, Yoon Y, Park C, Sohn J, Jeong J-J, Jeon B-N, Jang M, An C, Lee S, Kim YY, Kim G, Kim S, Kim Y, Lee GB, Lee EJ, Kim SG, Kim HS, Kim Y, Kim H, Yang H-S, Kim S, Kim S, Chung H, Moon MH, Nam MH, Kwon JY, Won S, Park J-S, Weinstock GM, Lee C, Yoon KW, Park H. 2021. Bifidobacterium bifidum strains synergize with immune checkpoint inhibitors to reduce tumour burden in mice. Nat Microbiol 6:277–288. doi:10.1038/s41564-020-00831-6. 33432149

[21] Morton JT, Marotz C, Washburne A, Silverman J, Zaramela LS, Edlund A, Zengler K, Knight R. 2019. Establishing microbial composition measurement standards with reference frames. Nat Commun 10:1–11. doi:10.1038/s41467-019-10656-5. 31222023 PMC6586903

[22] Yeoh YK, Zuo T, Lui GC-Y, Zhang F, Liu Q, Li AY, Chung AC, Cheung CP, Tso EY, Fung KS, Chan V, Ling L, Joynt G, Hui DS-C, Chow KM, Ng SSS, Li TC-M, Ng RW, Yip TC, Wong GL-H, Chan FK, Wong CK, Chan PK, Ng SC. 2021. Gut microbiota composition reflects disease severity and dysfunctional immune responses in patients with COVID-19. Gut 70:698–706. doi:10.1136/gutjnl-2020-323020. 33431578 PMC7804842

[23] Hazan S, Stollman N, Bozkurt HS, Dave S, Papoutsis AJ, Daniels J, Barrows BD, Quigley EM, Borody TJ. 2022. Lost microbes of COVID-19: Bifidobacterium, Faecalibacterium depletion and decreased microbiome diversity associated with SARS-CoV-2 infection severity. BMJ open Gastroenterology 9:e000871. doi:10.1136/bmjgast-2022-000871. PMC9051551 35483736

[24] Huang J, Liu D, Wang Y, Liu L, Li J, Yuan J, Jiang Z, Jiang Z, Hsiao WW, Liu H, Khan I, Xie Y, Wu J, Xie Y, Zhang Y, Fu Y, Liao J, Wang W, Lai H, Shi A, Cai J, Luo L, Li R, Yao X, Fan X, Wu Q, Liu Z, Yan P, Lu J, Yang M, Wang L, Cao Y, Wei H, Leung EL-H. 2022. Ginseng polysaccharides alter the gut microbiota and kynurenine/tryptophan ratio, potentiating the antitumour effect of antiprogrammed cell death 1/programmed cell death ligand 1 (anti-PD-1/PD-L1) immunotherapy. Gut 71:734–745. doi:10.1136/gutjnl-2020-321031. 34006584 PMC8921579

[25] Luu M, Riester Z, Baldrich A, Reichardt N, Yuille S, Busetti A, Klein M, Wempe A, Leister H, Raifer H, Picard F, Muhammad K, Ohl K, Romero R, Fischer F, Bauer CA, Huber M, Gress TM, Lauth M, Danhof S, Bopp T, Nerreter T, Mulder IE, Steinhoff U, Hudecek M, Visekruna A. 2021. Microbial short-chain fatty acids modulate CD8+ T cell responses and improve adoptive immunotherapy for cancer. Nat Commun 12:1–12. doi:10.1038/s41467-021-24331-1. 34210970 PMC8249424

[26] He Y, Fu L, Li Y, Wang W, Gong M, Zhang J, Dong X, Huang J, Wang Q, Mackay CR, Fu Y-X, Chen Y, Guo X. 2021. Gut microbial metabolites facilitate anticancer therapy efficacy by modulating cytotoxic CD8+ T cell immunity. Cell Metab 33:988–1000.e7. doi:10.1016/j.cmet.2021.03.002. 33761313

[27] Danne C, Sokol H. 2021. Butyrate, a new microbiota-dependent player in CD8+ T cells immunity and cancer therapy? Cell Rep Med 2:100328. doi:10.1016/j.xcrm.2021.100328. 34337557 PMC8324458

[28] Vétizou M, Pitt JM, Daillère R, Lepage P, Waldschmitt N, Flament C, Rusakiewicz S, Routy B, Roberti MP, Duong CPM, Poirier-Colame V, Roux A, Becharef S, Formenti S, Golden E, Cording S, Eberl G, Schlitzer A, Ginhoux F, Mani S, Yamazaki T, Jacquelot N, Enot DP, Bérard M, Nigou J, Opolon P, Eggermont A, Woerther P-L, Chachaty E, Chaput N, Robert C, Mateus C, Kroemer G, Raoult D, Boneca IG, Carbonnel F, Chamaillard M, Zitvogel L. 2015. Anticancer immunotherapy by CTLA-4 blockade relies on the gut microbiota. Science 350:1079–1084. doi:10.1126/science.aad1329. 26541610 PMC4721659

[29] Daillère R, Vétizou M, Waldschmitt N, Yamazaki T, Isnard C, Poirier-Colame V, Duong CPM, Flament C, Lepage P, Roberti MP, Routy B, Jacquelot N, Apetoh L, Becharef S, Rusakiewicz S, Langella P, Sokol H, Kroemer G, Enot D, Roux A, Eggermont A, Tartour E, Johannes L, Woerther P-L, Chachaty E, Soria J-C, Golden E, Formenti S, Plebanski M, Madondo M, Rosenstiel P, Raoult D, Cattoir V, Boneca IG, Chamaillard M, Zitvogel L. 2016. Enterococcus hirae and Barnesiella intestinihominis facilitate cyclophosphamide-induced therapeutic immunomodulatory effects. Immunity 45:931–943. doi:10.1016/j.immuni.2016.09.009. 27717798

[30] Pushalkar S, Hundeyin M, Daley D, Zambirinis CP, Kurz E, Mishra A, Mohan N, Aykut B, Usyk M, Torres LE, Werba G, Zhang K, Guo Y, Li Q, Akkad N, Lall S, Wadowski B, Gutierrez J, Kochen Rossi JA, Herzog JW, Diskin B, Torres-Hernandez A, Leinwand J, Wang W, Taunk PS, Savadkar S, Janal M, Saxena A, Li X, Cohen D, Sartor RB, Saxena D, Miller G. 2018. The pancreatic cancer microbiome promotes oncogenesis by induction of innate and adaptive immune suppression. Cancer discovery 8:403–416. doi:10.1158/2159-8290.CD-17-1134. 29567829 PMC6225783

[31] Griffin ME, Espinosa J, Becker JL, Luo JD, Carroll TS, Jha JK, Fanger GR, Hang HC. 2021. Enterococcus peptidoglycan remodeling promotes checkpoint inhibitor cancer immunotherapy. Science 373:1040–1046. doi:10.1126/science.abc9113. 34446607 PMC9503018

[32] Wolf AR, Wesener DA, Cheng J, Houston-Ludlam AN, Beller ZW, Hibberd MC, Giannone RJ, Peters SL, Hettich RL, Leyn SA, Rodionov DA, Osterman AL, Gordon JI. 2019. Bioremediation of a common product of food processing by a human gut bacterium. Cell host & Microbe 26:463–477.e8. doi:10.1016/j.chom.2019.09.001. 31585844 PMC6801109

[33] Bui TPN, Ritari J, Boeren S, De Waard P, Plugge CM, De Vos WM. 2015. Production of butyrate from lysine and the Amadori product fructoselysine by a human gut commensal. Nat Commun 6:1–10. doi:10.1038/ncomms10062. PMC4697335 26620920

[34] Rios-Covian D, Gueimonde M, Duncan SH, Flint HJ, de Los Reyes-Gavilan CG. 2015. Enhanced butyrate formation by cross-feeding between Faecalibacterium prausnitzii and Bifidobacterium adolescentis. FEMS microbiology Lett 362:fnv176. doi:10.1093/femsle/fnv176. 26420851

[35] Kim H, Jeong Y, Kang S, You HJ, Ji GE. 2020. Co-culture with Bifidobacterium catenulatum improves the growth, gut colonization, and butyrate production of Faecalibacterium prausnitzii: in vitro and in vivo studies. Microorganisms 8:788. doi:10.3390/microorganisms8050788. 32466189 PMC7285360

[36] Zaneveld J, McMinds R, Vega Thurber R, Thurber R. 2017. Stress and stability: applying the Anna Karenina principle to animal microbiomes. Nat Microbiol 2:17121. doi:10.1038/nmicrobiol.2017.121. 28836573

[37] Curtis MM, Hu Z, Klimko C, Narayanan S, Deberardinis R, Sperandio V. 2014. The gut commensal Bacteroides thetaiotaomicron exacerbates enteric infection through modification of the metabolic landscape. Cell host & Microbe 16:759–769. doi:10.1016/j.chom.2014.11.005. 25498343 PMC4269104

[38] Bäumler AJ, Sperandio V. 2016. Interactions between the microbiota and pathogenic bacteria in the gut. Nature 535:85–93. doi:10.1038/nature18849. 27383983 PMC5114849

[39] Goloshchapov OV, Olekhnovich EI, Sidorenko SV, Moiseev IS, Kucher MA, Fedorov DE, Pavlenko AV, Manolov AI, Gostev VV, Veselovsky VA, Klimina KM, Kostryukova ES, Bakin EA, Shvetcov AN, Gumbatova ED, Klementeva RV, Shcherbakov AA, Gorchakova MV, Egozcue JJ, Pawlowsky-Glahn V, Suvorova MA, Chukhlovin AB, Govorun VM, Ilina EN, Afanasyev BV. 2019. Long-term impact of fecal transplantation in healthy volunteers. BMC microbiology 19:1–13. doi:10.1186/s12866-019-1689-y. 31888470 PMC6938016

[40] Ott SJ, Waetzig GH, Rehman A, Moltzau-Anderson J, Bharti R, Grasis JA, Cassidy L, Tholey A, Fickenscher H, Seegert D, Rosenstiel P, Schreiber S. 2017. Efficacy of sterile fecal filtrate transfer for treating patients with Clostridium difficile infection. Gastroenterology 152:799–811.e7. doi:10.1053/j.gastro.2016.11.010. 27866880

[41] Sarrabayrouse G, Landolfi S, Pozuelo M, Willamil J, Varela E, Clark A, Campos D, Herrera C, Santiago A, Machiels K, Vermeire S, Martí M, Espin E, Manichanh C. 2020. Mucosal microbial load in Crohn’s disease: a potential predictor of response to faecal microbiota transplantation. EBioMedicine 51:102611. doi:10.1016/j.ebiom.2019.102611. 31901867 PMC6948165

[42] Sherry S, Xiao C, Durbrow K, Kimelman M, Rodarmer K, Shumway M, Yaschenko E. 2012. Ncbi sra toolkit technology for next generation sequence data. In Plant and Animal Genome XX Conference (January 14–18, 2012). Plant and Animal Genome.

[43] Bolger AM, Lohse M, Usadel B. 2014. Trimmomatic: a flexible trimmer for Illumina sequence data. Bioinformatics 30:2114–2120. doi:10.1093/bioinformatics/btu170. 24695404 PMC4103590

[44] Bushnell B. 2014. BBMap: a fast, accurate, splice-aware aligner. Lawrence Berkeley National Lab.(LBNL), Berkeley, CA (United States).

[45] Beghini F, McIver LJ, Blanco-Míguez A, Dubois L, Asnicar F, Maharjan S, Mailyan A, Manghi P, Scholz M, Thomas AM, Valles-Colomer M, Weingart G, Zhang Y, Zolfo M, Huttenhower C, Franzosa EA, Segata N. 2021. Integrating taxonomic, functional, and strain-level profiling of diverse microbial communities with bioBakery 3. Elife 10:e65088. doi:10.7554/eLife.65088. 33944776 PMC8096432

[46] Franzosa EA, McIver LJ, Rahnavard G, Thompson LR, Schirmer M, Weingart G, Lipson KS, Knight R, Caporaso JG, Segata N, Huttenhower C. 2018. Species-level functional profiling of metagenomes and metatranscriptomes. Nat Methods 15:962–968. doi:10.1038/s41592-018-0176-y. 30377376 PMC6235447

[47] Kanehisa M, Furumichi M, Tanabe M, Sato Y, Morishima K. 2017. KEGG: new perspectives on genomes, pathways, diseases and drugs. Nucleic acids Res 45:D353–D361. doi:10.1093/nar/gkw1092. 27899662 PMC5210567

[48] Fedarko MW, Martino C, Morton JT, González A, Rahman G, Marotz CA, Minich JJ, Allen EE, Knight R. 2020. Visualizing’omic feature rankings and log-ratios using Qurro. NAR genomics and Bioinformatics 2:lqaa023. doi:10.1093/nargab/lqaa023. 32391521 PMC7194218

[49] Bolyen E, Rideout JR, Dillon MR, Bokulich NA, Abnet CC, Al-Ghalith GA, Alexander H, Alm EJ, Arumugam M, Asnicar F, Bai Y, Bisanz JE, Bittinger K, Brejnrod A, Brislawn CJ, Brown CT, Callahan BJ, Caraballo-Rodríguez AM, Chase J, Cope EK, Da Silva R, Diener C, Dorrestein PC, Douglas GM, Durall DM, Duvallet C, Edwardson CF, Ernst M, Estaki M, Fouquier J, Gauglitz JM, Gibbons SM, Gibson DL, Gonzalez A, Gorlick K, Guo J, Hillmann B, Holmes S, Holste H, Huttenhower C, Huttley GA, Janssen S, Jarmusch AK, Jiang L, Kaehler BD, Kang KB, Keefe CR, Keim P, Kelley ST, Knights D, et al. 2019. Reproducible, interactive, scalable and extensible microbiome data science using QIIME 2. Nat Biotechnol 37:852–857. doi:10.1038/s41587-019-0209-9. 31341288 PMC7015180

[50] Wood DE, Lu J, Langmead B. 2019. Improved metagenomic analysis with Kraken 2. Genome Biol 20:1–13. doi:10.1186/s13059-019-1891-0. 31779668 PMC6883579

[51] Si B, Liang Y, Zhao J, Zhang Y, Liao X, Jin H, Liu H, Gu L. 2020. GGraph: an efficient structure-aware approach for iterative graph processing. IEEE Transactions on Big Data.

[52] Oksanen J, Blanchet FG, Kindt R, Legendre P, Minchin P, O’hara R, Simpson G, Solymos P, Stevens MHH, Wagner H, et al. 2013. Community ecology package. R package version 2 (0).

[53] Aitchison J. 1992. On criteria for measures of compositional difference. Mathematical Geology 24:365–379. doi:10.1007/BF00891269.

[54] Aitchison J, Pawlowsky-Glahn V. 1997. The one-hour course in compositional data analysis or compositional data analysis is simple. In Proceedings of IAMG 97:3–35.

[55] Coutzac C, Jouniaux J-M, Paci A, Schmidt J, Mallardo D, Seck A, Asvatourian V, Cassard L, Saulnier P, Lacroix L, Woerther P-L, Vozy A, Naigeon M, Nebot-Bral L, Desbois M, Simeone E, Mateus C, Boselli L, Grivel J, Soularue E, Lepage P, Carbonnel F, Ascierto PA, Robert C, Chaput N. 2020. Systemic short chain fatty acids limit antitumor effect of CTLA-4 blockade in hosts with cancer. Nat Commun 11:1–13. doi:10.1038/s41467-020-16079-x. 32358520 PMC7195489

[56] Mao J, Wang D, Long J, Yang X, Lin J, Song Y, Xie F, Xun Z, Wang Y, Wang Y, et al. 2021. Gut microbiome is associated with the clinical response to anti-PD-1 based immunotherapy in hepatobiliary cancers. J for Immunother Cancer 9. doi:10.1136/jitc-2021-003334. PMC8650503 34873013

[57] Heshiki Y, Vazquez-Uribe R, Li J, Ni Y, Quainoo S, Imamovic L, Li J, Sørensen M, Chow BKC, Weiss GJ, Xu A, Sommer MOA, Panagiotou G. 2020. Predictable modulation of cancer treatment outcomes by the gut microbiota. Microbiome 8:1–14. doi:10.1186/s40168-020-00811-2. 32138779 PMC7059390

[58] Usyk M, Pandey A, Hayes RB, Moran U, Pavlick A, Osman I, Weber JS, Ahn J. 2021. Bacteroides vulgatus and Bacteroides dorei predict immune-related adverse events in immune checkpoint blockade treatment of metastatic melanoma. Genome medicine 13:1–11. doi:10.1186/s13073-021-00974-z. 34641962 PMC8513370

[59] Derosa L, Routy B, Fidelle M, Iebba V, Alla L, Pasolli E, Segata N, Desnoyer A, Pietrantonio F, Ferrere G, Fahrner J-E, Le Chatellier E, Pons N, Galleron N, Roume H, Duong CP, Mondragón L, Iribarren K, Bonvalet M, Terrisse S, Rauber C, Goubet A-G, Daillère R, Lemaitre F, Reni A, Casu B, Alou MT, Alves Costa Silva C, Raoult D, Fizazi K, Escudier B, Kroemer G, Albiges L, Zitvogel L. 2020. Gut bacteria composition drives primary resistance to cancer immunotherapy in renal cell carcinoma patients. Eur urology 78:195–206. doi:10.1016/j.eururo.2020.04.044. 32376136

[60] Zheng Y, Wang T, Tu X, Huang Y, Zhang H, Tan D, Jiang W, Cai S, Zhao P, Song R, et al. 2019. Gut microbiome affects the response to anti-PD-1 immunotherapy in patients with hepatocellular carcinoma. J for Immunotherapy Cancer 7:1–7. 10.1186/s40425-019-0650-9 PMC6651993 31337439

[61] Fedorov D, Pavlenko A, Olekhnovich E, Klimina K, Pokataev I, Manolov A, Konanov D, Veselovsky V, Ilina E. 2021. Gut microbiome as a predictor of the anti-PD-1 therapy success: metagenomic data analysis. Biochemistry (Moscow). Suppl Ser B: Biomed Chem 15:161–165. 10.18097/PBMC20206606502 33372909

[62] Sun S, Luo L, Liang W, Yin Q, Guo J, Rush AM, Lv Z, Liang Q, Fischbach MA, Sonnenburg JL, Dodd D, Davis MM, Wang F. 2020. Bifidobacterium alters the gut microbiota and modulates the functional metabolism of T regulatory cells in the context of immune checkpoint blockade. Proc Natl Acad Sci USA 117:27509–27515. doi:10.1073/pnas.1921223117. 33077598 PMC7959554

[63] Jin Y, Dong H, Xia L, Yang Y, Zhu Y, Shen Y, Zheng H, Yao C, Wang Y, Lu S. 2019. The diversity of gut microbiome is associated with favorable responses to anti–programmed death 1 immunotherapy in Chinese patients with NSCLC. J thoracic Oncology 14:1378–1389. doi:10.1016/j.jtho.2019.04.007. 31026576

[64] Tanoue T, Morita S, Plichta DR, Skelly AN, Suda W, Sugiura Y, Narushima S, Vlamakis H, Motoo I, Sugita K, Shiota A, Takeshita K, Yasuma-Mitobe K, Riethmacher D, Kaisho T, Norman JM, Mucida D, Suematsu M, Yaguchi T, Bucci V, Inoue T, Kawakami Y, Olle B, Roberts B, Hattori M, Xavier RJ, Atarashi K, Honda K. 2019. A defined commensal consortium elicits CD8 T cells and anti-cancer immunity. Nature 565:600–605. doi:10.1038/s41586-019-0878-z. 30675064

