# Supplementary figures and images for: Consistent Stool Metagenomic Biomarkers Associated with the Response To Melanoma Immunotherapy
Source: mSystems. 2023 Feb 21;8(2):e01023-22. doi: 10.1128/msystems.01023-22 (PMC10134792; doi:10.1128/msystems.01023-22)

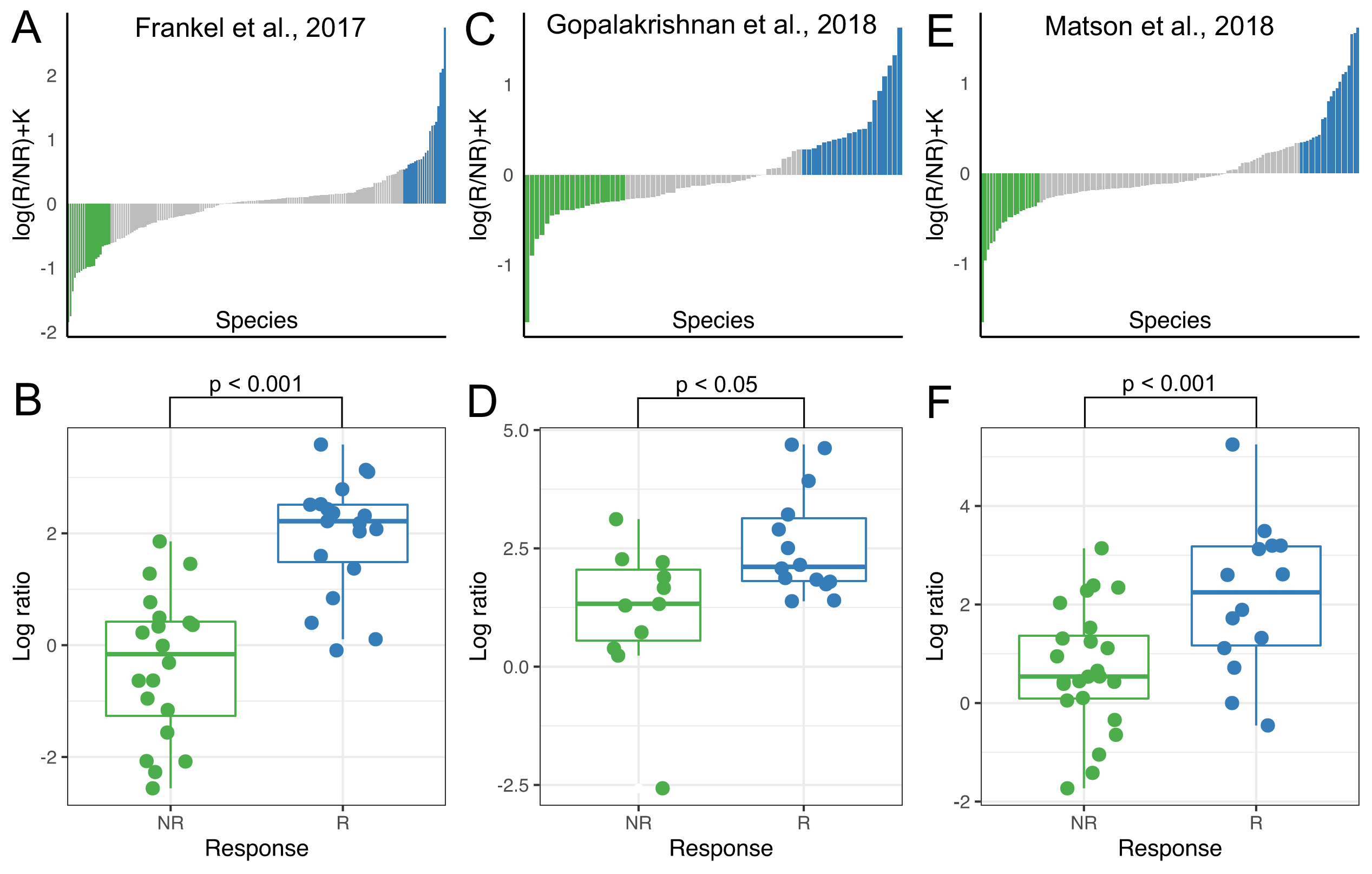

Supplement: FIG S1 [file msystems.01023-22-s0001.tif]

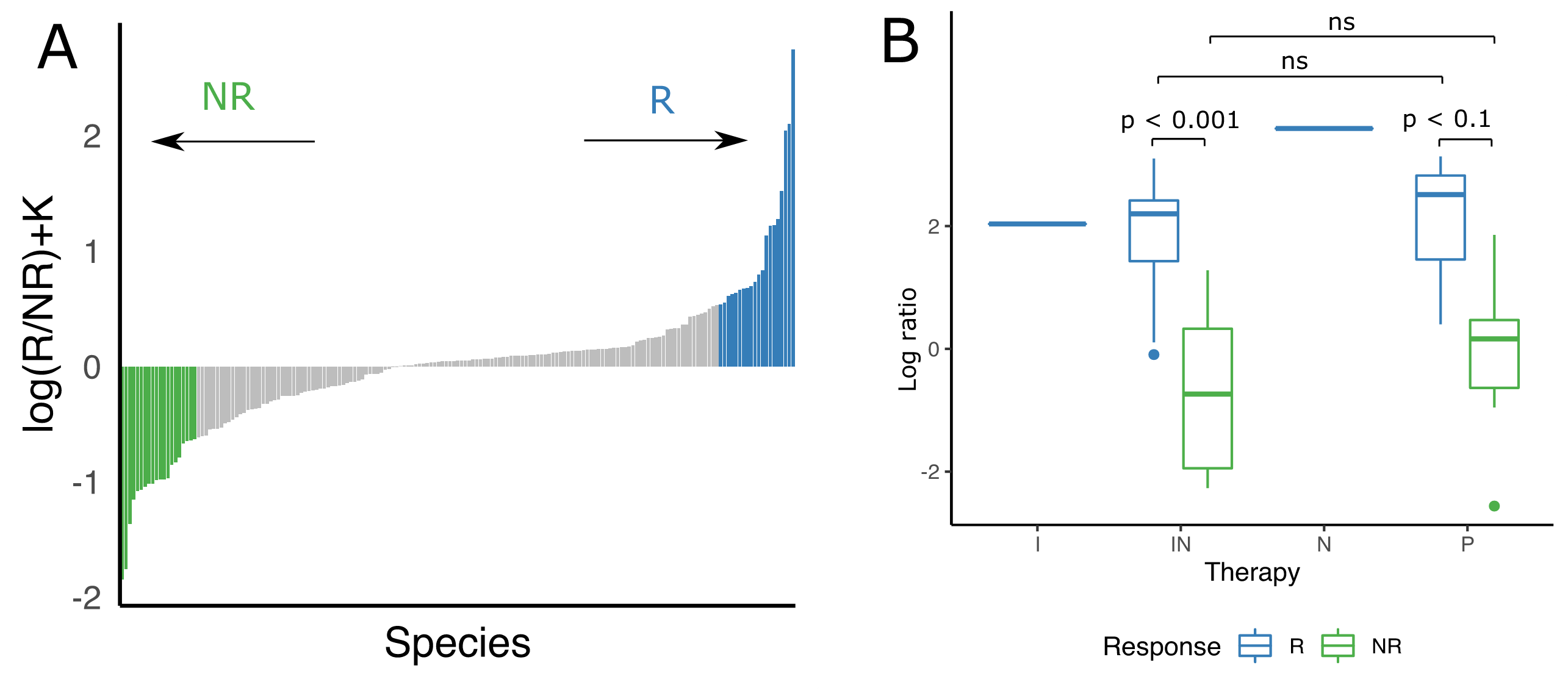

Supplement: FIG S2 [file msystems.01023-22-s0002.tif]

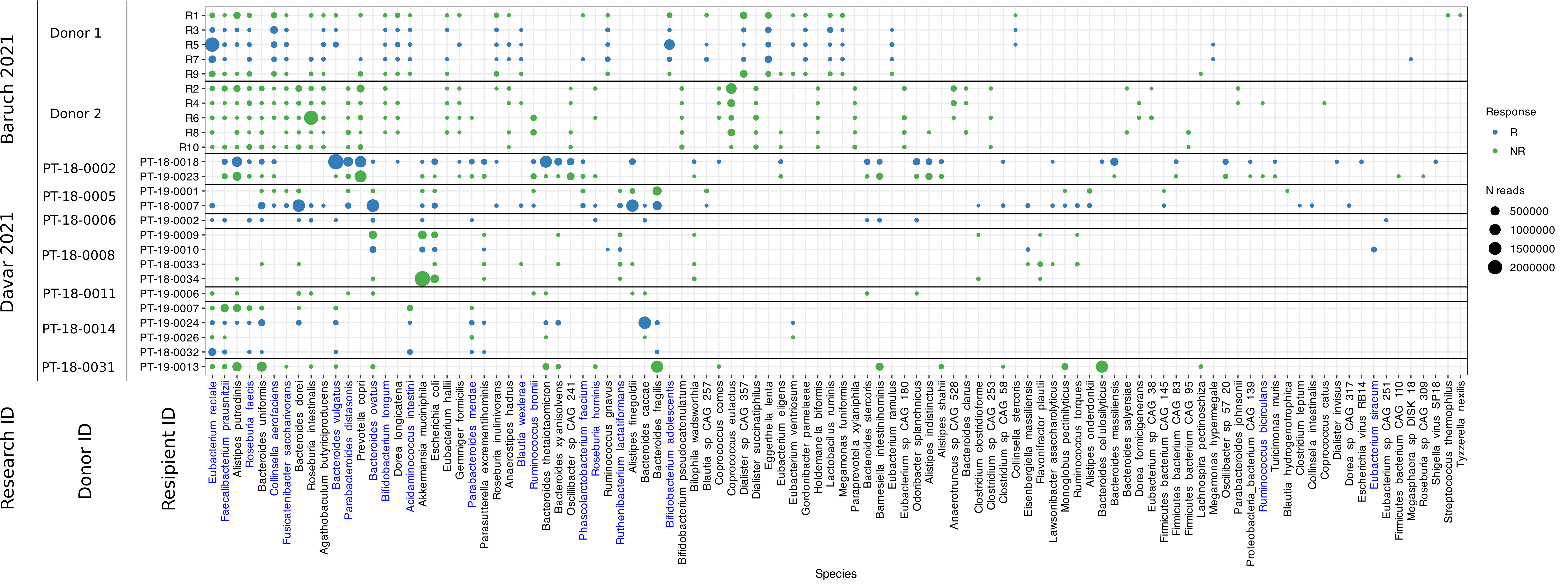

Supplement: FIG S3 [file msystems.01023-22-s0003.tif]

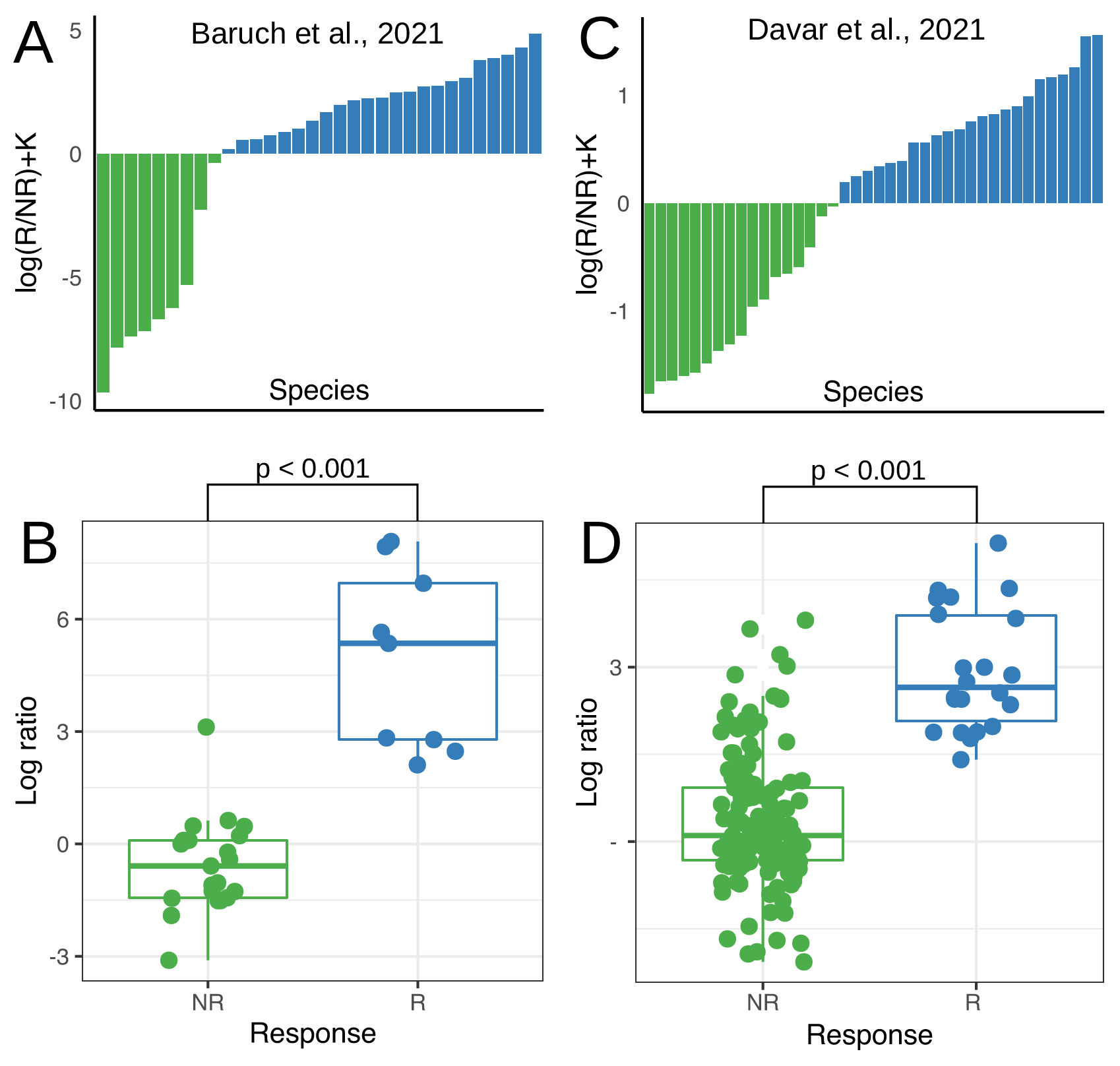

Supplement: FIG S4 [file msystems.01023-22-s0004.tif]
